# The roles of habitat isolation, landscape connectivity and host community in tick-borne pathogen ecology

**DOI:** 10.1098/rsos.240837

**Published:** 2024-11-06

**Authors:** Grace Shaw, Marie Lilly, Vincent Mai, Jacoby Clark, Shannon Summers, Kimetha Slater, Sandor Karpathy, Angie Nakano, Arielle Crews, Alexandra Lawrence, Jordan Salomon, Samantha Brianne Sambado, Andrea Swei

**Affiliations:** ^1^ Department of Biology, San Francisco State University, Holloway Avenue, San Francisco, CA 1600, USA; ^2^ Department of Ecology, Evolution and Environmental Biology, Columbia University, Amsterdam Avenue, New York, NY 1200, USA; ^3^ Division of Vector-Borne Diseases, National Center for Emerging and Zoonotic Infectious Diseases, Centers for Disease Control and Prevention, Rampart Road, Fort Collins, CO 80521, USA; ^4^ Division of Vector-Borne Diseases, National Center for Emerging and Zoonotic Infectious Diseases, Centers for Disease Control and Prevention, Clifton Road, Atlanta, GA 1600, USA; ^5^ San Mateo County Mosquito and Vector Control District, Rollins Rd, Burlingame, CA 1351, USA; ^6^ Division of Forestry and Natural Resources, West Virginia University, 322 Evansdale Dr Percival Hall, Morgantown, WV, USA; ^7^ Department of Veterinary Integrated Biosciences, Texas A&M University, College Station, TX, USA; ^8^ Department of Ecology, Evolution, and Marine Biology, University of California, Santa Barbara, CA, USA

**Keywords:** *Borrelia burgdorferi*, *Borrelia miyamotoi*, habitat fragmentation, island biogeography, *Ixodes pacificus*, *Rickettsia tillamookensis*

## Abstract

Habitat loss and forest fragmentation are often linked to increased pathogen transmission, but the extent to which habitat isolation and landscape connectivity affect disease dynamics through movement of disease vectors and reservoir hosts has not been well examined. Tick-borne diseases are the most prevalent vector-borne diseases in the United States and on the West Coast, *Ixodes pacificus* is one of the most epidemiologically important vectors. We investigated the impacts of habitat fragmentation on pathogens transmitted by *I. pacificus* and sought to disentangle the effects of wildlife communities and landscape metrics predictive of pathogen diversity, prevalence and distribution. We collected pathogen data for four co-occurring bacteria transmitted by *I. pacificus* and measured wildlife parameters. We also used spatial data and cost-distance analysis integrating expert opinions to assess landscape metrics of habitat fragmentation. We found that landscape metrics were significant predictors of tick density and pathogen prevalence. However, wildlife variables were essential when predicting the prevalence and distribution of pathogens reliant on wildlife reservoir hosts for maintenance. We found that landscape structure was an informative predictor of tick-borne pathogen richness in an urban matrix. Our work highlights the implications of large-scale land management on human disease risk.

## 1. Introduction

Rapid shifts in land use, such as landscape degradation for urbanization, mining or agriculture, have caused the emergence of many vector-borne zoonotic diseases [1–4]. The impact of habitat fragmentation on the emergence of vector-borne diseases has largely focussed on the impact of habitat patch size [5–7]. However, habitat isolation and distance to a founding host population are other aspects of island biogeography theory with relevance to pathogen richness [8,9] and are starting to be applied to other vector-borne disease systems [10–12].

Habitat fragmentation can have important implications for tick-borne disease dynamics [5,6,13,14]. Tick-borne pathogens are maintained by natural transmission cycles between a tick vector and reservoir hosts that serve as the tick’s bloodmeal. The presence, movement patterns and pathogen competency of key tick hosts, such as deer and rodents, influence pathogen prevalence and disease risk by carrying pathogens or attached ticks wherever they migrate and disperse [15–19]. In the western United States, the western blacklegged tick, *Ixodes pacificus,* vectors numerous tick-borne pathogens and is the vector for the causal bacteria of Lyme disease, *Borrelia burgdorferi sensu stricto* [20], among others. The presence of reservoir hosts such as dusky-footed woodrats (*Neotoma fuscipes*) and various *Peromyscus* species such as the deer mouse, California deer mouse, and pinyon mouse, is important for maintaining higher tick infection prevalence with *B. burgdorferi*, while greater predator diversity is linked with lower tick burdens on rodents, and therefore lower pathogen transmission rates between ticks and reservoir hosts [16,21]. Despite the established positive relationship between forest fragmentation, reforestation and Lyme disease risk in the northeastern United States [5,6], a generalized and mechanistic understanding of the relationship between habitat fragmentation, community composition and human disease risk is still lacking. The transmission mode of tick-borne pathogens also plays a crucial role in their maintenance and amplification within a disease system. Horizontally transmitted pathogens are pathogens that can only be acquired from a reservoir bloodmeal host while transovarially transmitted pathogens are transmitted from adult female ticks to their progeny and do not require a vertebrate reservoir [22]. While horizontally transmitted pathogens such as *B. burgdorferi sensu stricto*, one of the causative agents of Lyme disease and *Borrelia bissettiae* [23], a potential cause of borreliosis [24–26], have complex pathogen cycles often involving several reservoir hosts, pathogens capable of transovarial transmission can be less dependent on reservoir hosts to maintain the pathogen. However, reservoir hosts can still contribute to pathogen amplification and influence spatial disease risk of transovarially transmitted pathogens [27]. *Borrelia miyamotoi* [28] and *Rickettsia tillamookensis* [29] are two potentially emerging human pathogens carried by *I. pacificus* in California [29–31]. Evidence has shown *B. miyamotoi* to be capable of transovarial transmission while the transmission mode of *R. tillamookensis* is still unknown [27,32]. Investigating the distribution of these four pathogens offers an opportunity to address whether pathogen presence is mediated by the combined impacts of landscape ecology and transmission mode of vector-borne diseases. By studying a suite of pathogens, we also sought to understand the circumstances under which surveillance of a focal pathogen may be misleading to overall disease risk.

During their blood meals, ticks remain attached to their host for several days and can leave the host with ongoing pathogenic infections. Therefore, when modelling the distribution of tick-borne diseases, it is crucial to consider the movement of hosts that transport ticks and the pathogens they vector. Such hosts include rodents, small mammals, deer, meso-carnivores and large predators, which all play important but variable roles in tick life cycles or predator–prey relationships that drive blood-meal availability. The absence of key species in these communities can also create trophic disruptions, leading to changes in abundance of prey species, prey switching or behavioural changes among species in the community facing increased or decreased competition. In turn, these cascading effects shift the availability of tick blood meals [33,34]. For tick-borne diseases, habitat isolation may therefore be better described by the habitat connectivity of reservoir host species rather than the simpler metric of Euclidean distance between habitats [35,36]. We modelled least-cost paths between habitat fragments and the nearest remaining intact habitat by assigning friction values to different landscape elements based on the likelihood tick hosts will use each element for movement [37]. Although cost-distance modelling has proved to be a flexible tool in evaluating functional connectivity, few studies have looked at the relationship between habitat connectivity and tick-borne pathogens in urban areas [11,12,38], and none have considered the dispersal behaviour of an entire mammalian community. Cost distance was chosen for this study as it remains the simplest connectivity algorithm amongst other popular approaches such as Circuitscape and resistant kernels, and error in the exact pathway of dispersal is acceptable as only the relative connectivity levels amongst habitat fragments are in question [39–42].

Here, we evaluate the impact of landscape heterogeneity and habitat isolation on tick-borne pathogens with different transmission modes, particularly those with varying levels of reliance on hosts that can transmit and amplify the pathogens. We ask: (i) is there a relationship between habitat isolation and tick-borne disease risk indicators such as the density of ticks, density of infected ticks, pathogen prevalence, tick infection status and overall pathogen richness? and (ia) if there is a relationship, is habitat connectivity a better predictor than isolation distance? (ii) what combination of landscape and wildlife variables best predicts these tick-borne disease risk indicators? and finally (iii) how does the transmission mode of tick-borne pathogens interact with the variables predictive of individual pathogen prevalence and tick infection status? Understanding the landscape and wildlife patterns driving the distribution of this multi-pathogen disease system will help predict distribution and diversity patterns in other vector-borne pathogen communities.

## 2. Material and methods

### 2.1. Field sites and sampling

Field sampling for ticks took place at 19 sites (electronic supplementary material, table S1) along a fragmentation gradient in north coastal California from 2016 to 2021 as described in Lawrence *et al.* [43] and Salomon *et al.* [21]. Landscape components that were considered to contribute to fragmentation during the selection of field sites included adjacent housing, city scape, multi-lane roads or highways, human-designed green spaces such as golf courses, bodies of water such as lakes, reservoirs and bays, and large swaths of impervious landscape such as car parks. Our most fragmented sites included urban parks completely surrounded by housing, while the least fragmented sites were open space preserves included within larger complexes of natural area, equating to several thousand acres of contiguous green space. At 14 of the 19 sites, wildlife surveys were conducted, including rodent live trapping to determine the relative rodent species richness and abundance, and wildlife camera traps to measure the relative richness and a relative count index of larger vertebrates. All surveys took place within the peak *I. pacificus* nymphal season (March–June) on 0.5 ha plots with a 7 × 7 grid array as previously described [43]. Rodents were captured with two Sherman traps (7.6 × 9.5 × 30.5 cm; H.B. Sherman Traps, Tallahassee, FL, USA) at each trapping station over a three-night trapping window. All captured animals were processed as described previously, which included identifying the species, age and sex, and marking each individual with an ear tag upon first capture to identify re-captured individuals during the second and third trapping nights [21]. Two motion sensor wildlife cameras (Bushnell models nos. 119736, 119836, 119836C, Bushnell, KS, USA) were positioned approximately one metre above the ground, on trees, facing opposite directions towards open areas or game trails, capturing 40 days and nights of wildlife activity, similarly to Lawrence *et al.* [43], though earlier studies used only one camera per site. Animals in the photographs were identified to species level by field biologists using visual observations. Questing ticks were collected twice per season via standardized tick drags at each plot’s 70.8 m transects, for a total of 995 m^2^ each season. Drag cloths were constructed out of heavy white flannel material and were a standard 1 x 1 m size [44]. Ticks were identified in the laboratory to species, sex and life stage [45,46], and densities were measured as total *I. pacificus* nymphs collected per 995 m^2^ (San Francisco State University IACUC protocols nos. AU16-05R1a and AU19-01R2, California Department of Fish & Wildlife collection permits SC-8407 and S-203370009).

The relative abundances or count indices of rodents and large vertebrates were measured separately via mark-recapture live trapping and wildlife camera data as described by Lawrence *et al.* [43] and Salomon *et al.* [21]. Relative rodent abundance was calculated for each year and site using the R package ‘Rcapture’ [47]. We then used the R package, ‘vegan’, to calculate the relative Shannon diversity index for both variables across each year of sampling at each site [48]. All rodent and wildlife variables (abundance, count index, richness and diversity) were measured as relative metrics between sites and should not be considered absolute measurements.

### 2.2. Molecular analysis

We extracted DNA from whole *I. pacificus* nymphs using the Qiagen DNeasy Blood and Tissue Kit (Qiagen, Valencia, California, USA) with a few modifications; the tick surface was decontaminated with 70% ethanol prior to beginning the extraction, ticks were crushed using a disposable pestle, and DNA was eluted into 100 μl of elution buffer in the final step to increase DNA concentration [27,43]. Three separate polymerase chain reaction (PCR) assays and gene targets were used to test for the presence or absence of *B. burgdorferi sensu stricto* (s.s.), *B. bissettiae*, *B. miyamotoi* and *R. tillamookensis* within questing nymphal *I. pacificus*. A nested 5 S-23S rDNA PCR protocol [49] was first used to test for the *B. burgdorferi sensu lato* (s.l.) complex [50]. Positive sequences were then edited and aligned with Geneious v 11.15 and compared with sequences in NCBI BLAST to confirm their identity [50] (https://blast.ncbi.nlm.nih.gov/Blast.cgi). A 16 S-23S rDNA quantitative PCR (qPCR) protocol was then used to test for *B. miyamotoi* [51] and a qPCR assay targeting the aspS tRNA ligase gene was used to test for *R. tillamookensis* [52]. Samples were tested in triplicate and run with one positive control per pathogen (*B. burgdorferi* isolate CA4, *B. miyamotoi* isolate HT31 and *R. tillamookensis* strain Tillamook 23) and a minimum of three negatives (ultraviolet sterilized PCR grade water) per run.

### 2.3. Landscape fragmentation analysis

We measured three categories of fragmentation variables: site composition, nearby habitat and isolation/connectivity to intact habitat (electronic supplementary material, table S2). Site composition metrics include habitat patch size, perimeter length and edginess (perimeter/area) (electronic supplementary material, methods; Landscape fragmentation metrics). Nearby habitat was measured by calculating the surrounding green space within a 1 km radius of each plot, ignoring any green space that continued beyond this buffer. One kilometre was chosen for the buffer distance to align with standards of a similar ‘proximity index’ measurement and to remain consistent with previous tick ecology studies [43,53] (electronic supplementary material, methods; Landscape fragmentation metrics). Focusing on vegetation within 1 km also provides a landscape metric pertinent to the important small-bodied hosts in this system, those that do not migrate or disperse long distances but who host juvenile tick stages and carry *B. burgdorferi,* such as rodent species found in our study sites [21,54,55]. For the surrounding green space calculations, a 30 × 30 m cell size was used. This choice assumes that vegetation which fills less than half of one cell (and therefore would not classify the cell as vegetative) will not be as attractive for use by our focal species owing to edge effects, increases predation risk, or resource limitations and therefore is not considered in the summation of green space.

We measured isolation and connectivity to intact habitat in two ways: Euclidean distance and functional connectivity (electronic supplementary material, methods; Cost-distance analysis). Euclidean distance, the simplest measurement of habitat isolation, was included to determine the use of rudimental isolation metrics in disease systems when more sophisticated analyses are not available or cumbersome to perform. Functional connectivity measures the permeability of the matrix between habitat patches for the target species (i.e. tick and pathogen hosts: rodents, small mammals, deer, meso-carnivores and large predators). The functional connectivity, measured as cost-distance, used consolidated expert opinion data from 30 wildlife biologists on the friction of land cover types represented in the California Department of Forestry and Fire Protection’s fveg dataset and the highway category from the U.S. Census Bureau’s TIGER/line shapefiles and the California Department of Transportation’s National Highway System dataset (CALFIRE-FRAP and California Department of Forestry and Fire Protection 2015; United States Census Bureau 2021; California Department of Transportation 2022). Friction levels were recorded separately for each of 15 focal species or species groups in the study system, chosen because they are known or suspected pathogen or *I. pacificus* hosts (*Canis latrans, Didelphis virginiana, Lepus californicus, Lynx rufus, Mephitis mephitis, Neotoma fuscipes, Odocoileus hemionus, Otospermophilus* sp*., Peromyscus* sp*., Procyon lotor, Puma concolor, Sciurus* sp*., Sylvilagus bachmani, Urocyon cinereoargenteus, Vulpes vulpes*) [54,56–65]. These values were then aggregated as deer, predator, and overall wildlife friction level (electronic supplementary material, methods; Cost-distance analysis).

For our study system, we defined intact habitats in two ways: (i) intact natural habitat (i.e. conifer, hardwood, herbaceous, shrub and/or wetland); and (ii) intact forests (i.e. only conifer and/or hardwood). With our first definition of intact habitat, we seek to understand the landscape-level impacts of connectivity to those wildlife communities that are likely to retain top predators with large home range size requirements, and therefore at low risk for trophic disruptions and predatory release, both of which have been linked to patterns of tick-borne disease emergence across North America [33,66]. Because our analysis looked at habitat suitability and dispersal for an entire mammalian community, and many of the focal species involved in the black-legged tick life cycle are habitat generalists (such as deer and mountain lions) [67, 68], only habitats restrictive to the majority of our focal species were excluded. These areas include land cover types classified as agriculture, barren/other, desert (though no desert is present in our study system), urban, water and barriers such as major roads or highways. Our second definition of intact habitat is more restrictive and only looks at the impacts of connectivity to wildlife communities in areas most suitable for *I. pacificus* (i.e. forests), which need the protective cover provided by leaf litter to avoid desiccation and temperature extremes during the juvenile life stages [69,70].

Both categories of intact habitat were only considered ‘intact’ habitats if they were larger than the home range size of all wildlife in our study system. An area of 11 333 ha was used as a proxy for this size requirement because this was the smallest home range size recorded for any individual *Puma concolor* in coastal California, which has the largest home range size of any animal in our study area [71]. By only including habitats large enough to host the full range of California wildlife, we have filtered our intact habitats for areas most likely to represent pre-urbanization community dynamics.

### 2.4. Statistical analysis

To determine the relationship between habitat fragmentation and the presence of zoonotic tick-borne pathogens, we constructed generalized linear mixed-effects models (GLMMs) using R studio (Version 1.4) and the ‘glmmTMB’ package [72]. Our models integrated both landscape and wildlife variables as predictors for pathogen risk at each habitat patch. Models predicting the density of ticks or infected ticks used negative binomial error distributions, the pathogen richness model used a Poisson error distribution and models analysing pathogen presence had binomial or beta error distributions. Landscape metrics considered as fixed effects in these models included habitat patch size, perimeter length, edginess (perimeter/area), surrounding green space, Euclidean distance to intact habitat, Euclidean distance to intact forest, and host, predator and deer cost-distance to intact habitat and forests (electronic supplementary material, table S2). Wildlife fixed effects included the relative richness and Shannon diversity of rodents, predators, and the overall wildlife community, the relative abundance for rodents and relative count index for wildlife.

Most Lyme disease infections are transmitted by nymphs and therefore we focussed on this life stage for our models [73]. Response variables were chosen based on previous literature demonstrating the link between human disease risk and nymphal tick densities, density of infected nymphs and infection prevalence among nymphs [15,74].

We created models for the density of *I. pacificus* nymphs (DON) (count per site, per year), density of *B. burgdorferi* s.l. infected nymphs (DIN) (count per site, per year), pathogen richness for our pathogens of interest (*B. burgdorferi* s.s., *B. bissettiae*, *B. miyamotoi* and *R. tillamookensis*), *B. burgdorferi* s.l. nymphal infection prevalence (NIP) (proportion), as well as the nymphal infection status (NIS) (binary outcome; 1 = infected, 0 = uninfected) of *B. burgdorferi* s.l., *R. tillamookensis* and *B. miyamotoi* (electronic supplementary material, table S3). Models for *B. burgdorferi* s.l. DIN, NIP and NIS were used to better represent Lyme disease risk from both *B. burgdorferi* s.s. and *B. bissettiae*. However, *B. burgdorferi* s.s. and *B. bissettiae* were represented separately in the pathogen richness model to best highlight in which cases both species, only one, or neither of these related pathogens are present. We chose to create a binomial infection status model (NIS, binary outcome) in addition to our infection prevalence (NIP) model to give more power to the dataset by correlating site variables with each sample. The use of NIS models also allowed us to compare model predictors for the two transmission modes (vertically vs. non-vertically transmitted) represented by our four pathogens of interest, as only the most common pathogens could be modelled with aggregated NIP data. *Borrelia burgdorferi* s.s. and *B. bissetiae* are examples of non-vertically transmitted pathogens, *B. miyamotoi* represents a vertical transmission example and *R. tillamookensis* is an example of a pathogen with unknown vertical transmission capability. Wildlife model parameters were lagged such that wildlife Shannon diversity, predator Shannon diversity, rodent Shannon diversity and rodent species richness were correlated with tick response variables the following year (*t+*1) because wildlife abundance drives nymphal tick populations in the following season [75].

The best-fit models were selected based on the lowest Akaike information criterion (AIC) score of any combination of predictor variables. Predictor variables with non-significant *p*-values were only included if they brought the AIC score down by at least two points from a model with fewer variables [76]. In some instances, to use data from years where only some predictor variables were available, separate models were run and adjusted with a Bonferroni correction to account for multiple analyses. For example, our wildlife parameters are associated with lagged DON and DIN and therefore incorporate fewer seasons of data to account for the beginning of our study when we did not have data from the prior year’s wildlife community, and our 2020 season in which we could not measure wildlife variables at our sites owing to pandemic related restrictions on fieldwork. In the DON model, we used a Bonferroni correction to examine two predictor variables in separate models because they had a high covariance with one another. Our DON and DIN datasets followed a negative binomial distribution pattern, our NIP model was best fit by a beta distribution, our binary NIS model required a binomial distribution, and our pathogen model used a Poisson distribution. We specified zero-inflated distributions for models of count or proportion data such as DON, DIN and NIP to account for response datasets with many zeros. All models included site and year as random effects.

## 3. Results

Between 2016 and 2021, we drag sampled 137 310 m^2^ across 19 sites, collected more than 2407 *I. pacificus* nymphs, and tested 1805 nymphs for *B. burgdorferi* s.s., and *B. bissettiae*. When we could not process all nymphs collected (i.e. high tick density years), similar numbers of ticks were tested for all sites, except for low-density sites from which we tested all nymphs (electronic supplementary material, table S3). In addition, we tested 955 *I. pacificus* nymphs for *B. miyamotoi* (collected during 2016, 2018 and 2021) and 1012 nymphs for *R. tillamookensis* (collected between 2016 and 2021). When comparing across pathogens, we only used data from collections that were tested for all four pathogens with similar effort, totalling in 667 nymphs. We collected more than 200 days of wildlife camera trap data across five nymphal tick seasons (2016–2019 and 2021) and recorded 12 large vertebrate species. Additionally, we trapped 1647 rodents from 10 taxon (electronic supplementary material, results). Wildlife metrics were generated at 14 of our 19 sites, which included relative rodent diversity, predator diversity and total mammalian diversity (including animals captured with live traps and our wildlife cameras). Five of our sample sites lacked wildlife data owing to limited staffing capacity for camera and rodent trapping at these sites and were excluded from analyses of wildlife predictor variables.

**Figure 1.**
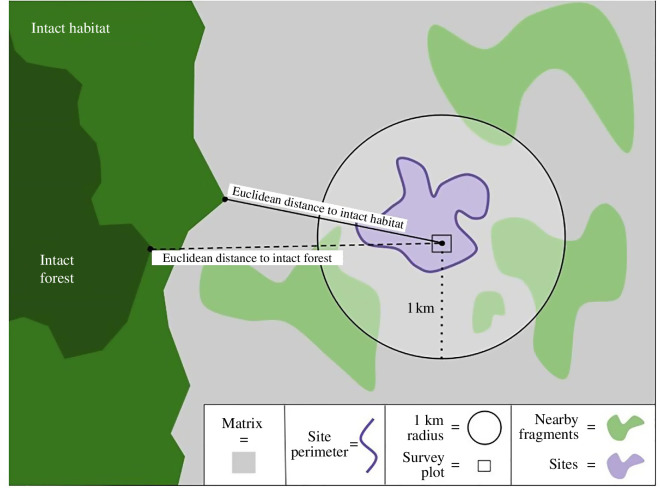
Landscape metrics used as predictor variables in our models. Surrounding green space measures the total natural area within a 1 km radius of the survey plot (m^2^).

For all 19 of our study sites, we measured the habitat patch size, perimeter length, edginess (perimeter to area ratio), surrounding green space, Euclidean and cost-distance to the nearest intact habitat (conifer, hardwood, herbaceous, shruband/or wetland), and Euclidean and cost-distance to the nearest intact forest (conifer and/or hardwood) (figure 1). We found that for all models except our *B. miyamotoi* NIS model, the cost-distance calculations proved similarly, or in many cases, slightly less predictive of our response variables than the more straightforward Euclidean distance measurements. For example, host community cost-distance to intact habitat and Euclidean distance to intact habitat had similar estimates for our *R. tillamookensis* NIS model; however, Euclidean distance was the better predictor (estimate of −0.58 vs. −0.50).

### 3.1. *Borrelia burgdorferi sensu stricto* prevalence was highest among pathogens

Of our four bacteria of interest*, B. burgdorferi* s.s. had the highest infection prevalence among questing *I. pacificus* nymphs across all sites and years sampled (minimum average of 5.1% infection prevalence after the exclusion of a 100% infection prevalence at Worcester Park in 2018 when only one nymph was collected and tested positive for *B. burgdorferi* s.s.; electronic supplementary material, table S4). *Borrelia miyamotoi* was the next most prevalent (1.9%), followed by *R. tillamookensis* (1.8%). *Borrelia bissettiae* had an average infection prevalence between 0.1 and 0.9%, depending on the identity of several samples that were PCR positive for *B. burgdorferi* s.l. but did not sequence to the species level (electronic supplementary material, table S4).

### 3.2. Higher density of *Ixodes pacificus* nymphs in large, minimally isolated habitats with adjacent green space

**Figure 2.**
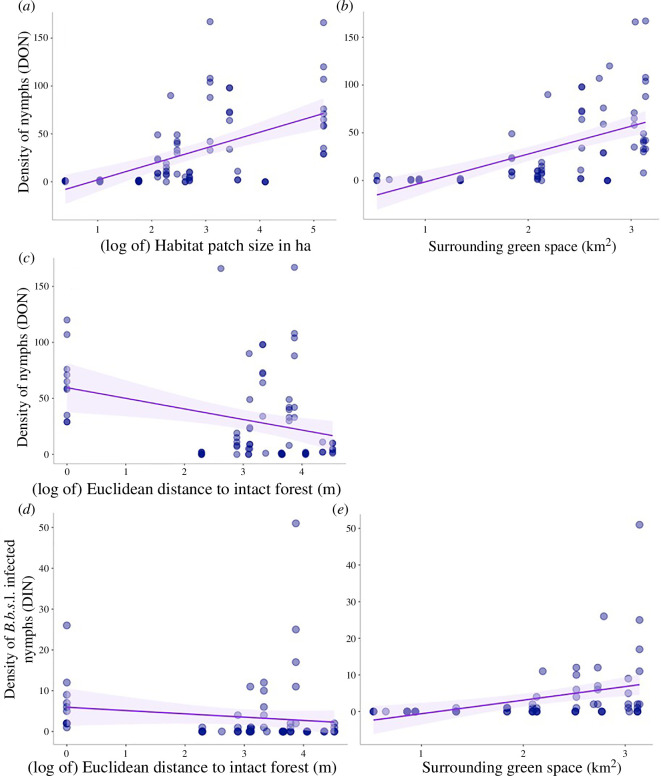
(*a*–*e*) Northern California. Density of *I. pacificus* nymphs (DON) was positively correlated with habitat patch size and the surrounding green space, and negatively correlated with Euclidean distance to intact forests. Density of *Borrelia burgdorferi* s.l. (*B.b*.s.l.) infected *I. pacificus* nymphs (DIN) was negatively correlated with Euclidean distance to intact forests, but positively correlated with surrounding green space.

Our DON model was best represented by two separate zero-inflated negative binomial GLMMs with a Bonferroni correction applied to account for multiple models used for variables with strong covariance (table 1). Density of nymphs increased with increasing habitat patch size and natural surrounding area and decreased as Euclidean distance from intact forests increased (figure 2*a–e*).

**Table 1.** Northern California. *Ixodes pacificus* disease models. (GLMMs for DON, *B. burgdorferi* s.l. (*B.b*.s.l.) DIN, and *B*.*b*.s.l. NIP with fragmentation metrics as predictor variables. Double lines indicate two separate models that were selected to predict the same response variable, with a Bonferroni correction applied. Landscape variables are in regular font while the wildlife predictors are italicized. The DON and DIN models had a zero-inflated negative binomial distribution, while the NIP model used a zero-inflated beta distribution. Site and year are included as random effects. The DON models include data from 2016 to 2021, the landscape DIN model includes infection data from 2016, 2018, 2019, 2020 and 2021, and the wildlife DIN and NIP models used data from 2018, 2019 and 2020 as nymphal infection was associated with the wildlife parameters from the previous season, and we were not able to collect wildlife data in 2020. The level of significance is denoted with asterisks for each predictor (***p‐value < 0.001; **p‐value < 0.01; *p‐value < 0.05).)

variable		estimate	s.e.	*z*-value	*p*‐value
**density of nymphs (DON**)					
	(log of) habitat patch size	0.24	0.20	1.23	0.2200
	surrounding green space	1.62	0.35	4.60	4.36e−06 ***
					
	(log of) Euclidean distance to intact forests	−0.63	0.28	−2.24	0.0254 *
**density of infected nymphs (DIN)*—B.b.*s.l**.					
	(log of) habitat patch size	−1.02	0.72	−1.40	0.1603
	surrounding green space	3.11	1.21	2.58	0.0099 **
	(log of) Euclidean distance to intact forests	−0.93	0.47	−1.98	0.0478 *
					
(**lagged) density of infected nymphs (DIN)*—B.b.*s.l**.					
	(*relative) predator Shannon diversity*	1.01	0.41	2.49	0.0128 *
(**lagged) nymphal Infection prevalence (NIP)— *B.b.*s.l**.					
	(*relative) wildlife Shannon diversity*	3.44	0.13	27.52	<2e−16 ***
	(*relative) rodent Shannon diversity*	1.46	0.10	14.75	<2e−16 ***

In sites smaller than 100 ha (2.5 ha, 11 ha, and two sites at 57 ha), we collected a maximum of two *I. pacificus* nymphs per 995 m^2^ dragged (average of 0.07 nymphs per 100 m^2^ across all sampling years), whereas in intermediate sized sites (130 and 188 ha, respectively) we found an average of 20 and 10 nymphs per drag (average of 1.49 nymphs per 100 m^2^ across all sampling years). However, some sites up to 13 000 ha also yielded zero *I. pacificus* nymphs (depending on the year).

We observed that almost any level of isolation distance could result in zero *I. pacificus* nymphal density years. For example, the site with our minimum isolation distance of 0.2 km (Worcester Park) had an average of only 0.83 nymphs 995 m^2^ dragged, though this site was also one of our smallest at 11 ha so it is difficult to dissect the impacts of patch size and isolation. The only sites that consistently contained *I. pacificus* nymphs were those within the bounds of intact habitat, and therefore had an isolation distance of zero.

### 3.3. Density of *Borrelia burgdorferi sensu lato* infected nymphs decreases in large fragments, increases with predator Shannon diversity

DIN decreased with increasing habitat patch size. The surrounding green space and Euclidean distance to forests, however, maintained the same relationships as in our DON model where infected nymphs increased with increasing surrounding green space and decreased as the Euclidean distance increased. Predator diversity was also positively associated with DIN in a separate model (table 1; figure 2*a–e*).

Virtually no *B. burgdorferi* s.l. infected *I. pacificus* nymphs were collected at sites with less than 2 km^2^ of surrounding green space (a maximum of one infected nymph per site, per year). However, occurrences of zero infection prevalence occurred even in sites with the highest levels of surrounding green space.

### 3.4. *Borrelia burgdorferi sensu lato* nymphal infection prevalence and nymphal infection status correlated with wildlife predictors

**Figure 3.**
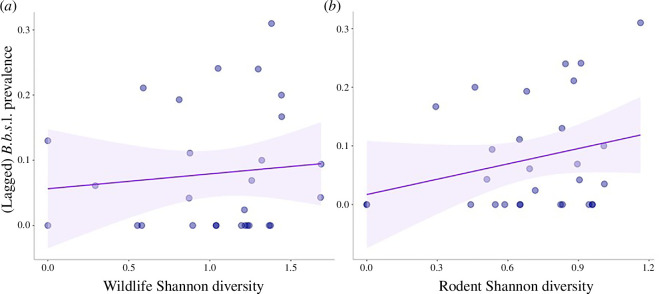
(*a–b*) Northern California. Wildlife and rodent Shannon diversity were the only predictive metrics for *Borrelia burgdorferi* s.l. (*B.b*.s.l.) nymphal infection prevalence (NIP). Wildlife diversity was measured as the relative count index of large vertebrates captured with wildlife camera traps, and rodent diversity was measured with a live trapping mark and recapture study. Both variables were positively correlated with *Borrelia burgdorferi* s.l. prevalence amongst *I. pacificus* nymphs, lagged by 1 year.

Lagged *B. burgdorferi* s.l. NIP and NIS were both positively correlated with wildlife variables, but no landscape variables (tables 1 and 2). Rodent and overall wildlife Shannon diversity were predictive of *B. burgdorferi* s.l. NIP, while rodent richness (range of 0–5) had the strongest association with *B. burgdorferi* s.l. infection status (figure 3*a–b*; electronic supplementary material, S1). One data point was removed because the sample size was too low.

**Table 2.** Northern California. *Ixodes pacificus* disease models. (GLMMs for *B. burgdorferi* s.l.*, R. tillamookensis* and *B. miyamotoi* NIS, with fragmentation metrics as predictor variables. The *B. burgdorferi* s.l. model only includes a wildlife variable and therefore uses lagged infection statuses, while the *R. tillamookensis* and *B. miyamotoi* models only incorporate a landscape variable and therefore are not lagged. The landscape variables are shown in regular font while the wildlife predictors are italicized. These models had zero-inflated binomial distributions. Site and year were included as random effects. The *B. burgdorferi* s.l. NIS model includes infection data from 2018, 2019, 2020, the *R. tillamookensis* model includes data from 2016, 2018, 2019, 2020 and 2021, and the *B. miyamotoi* model uses data from 2018 and 2021 owing to testing capacity constraints in other years. The level of significance is denoted with asterisks for each predictor (**p‐value < 0.01; *p‐value < 0.05).)

variable	estimate	s.e.	*z*-value	*p*‐value
(**lagged) nymphal infection status (NIS)*—B. burgdorferi* s.l**.				
(*relative) rodent richness*	0.61	0.23	2.72	0.0065**
**Nymphal infection status (NIS)*—R. tillamookensis***				
(log of) Euclidean distance to intact habitats	−0.58	0.32	−1.81	0.0708
**Nymphal infection status (NIS)*—B. miyamotoi***				
(log of) host community cost-distance to intact forest	−0.19	0.10	−1.99	0.0466*

The pinyon mouse (*Peromyscus truei*) was present at all *B. burgdorferi* s.l. nymphal infection sites and years except for the 2018 seasons at Tiburon Uplands Preserve and Worcester Park. Instead, rodents present at these sites included the dusky-footed woodrat (*Neotoma fuscipes*) and the California vole (*Microtus californicus*) at both sites, the deer mouse (*Peromyscus maniculatus*) at Tiburon Uplands Preserve, and the California deer mouse (*Peromyscus californicus*) and the western harvest mouse (*Reithrodontomys megalotis*) at Worcester Park. *Peromyscus truei* was also the only rodent present at some *B. burgdorferi* s.l. nymphal infection sites, though the species was also present at sites with years of zero *B. burgdorferi* s.l. infections.

### 3.5. Pathogen richness increases with more surrounding green space and shorter distance to intact habitat

**Figure 4.**
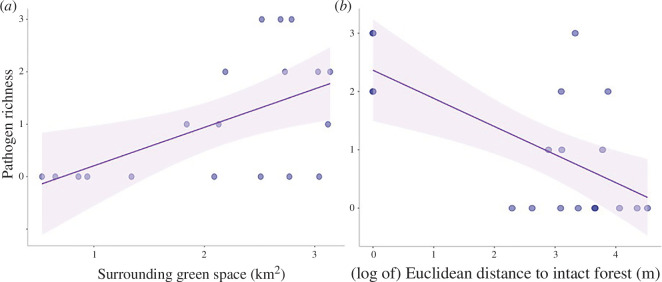
(*a–b*) Northern California. *Ixodes pacificus* tick-borne pathogen richness was positively correlated with surrounding green space, and negatively correlated with Euclidean distance to intact forests. This data only includes sampling carried out in 2021, as this was the most intensive sampling year for all four bacteria of interest.

Pathogen richness ranged from 0 to 3 (no sites were found to host all four bacteria of interest). GLMM analysis found richness to be positively correlated with the surrounding green space and negatively correlated with Euclidean distance to intact forests (table 3; figure 4*a,b*). Three sites had three pathogens occurring at the same time and represent our high pathogen richness sites (electronic supplementary material, table S5).

**Table 3.** Northern California. *Ixodes pacificus* disease models. (GLMM for pathogen richness with fragmentation metrics as predictor variables. This model had a zero-inflated Poisson distribution. The model only includes data from 2021 as this was the only year we had complete pathogen surveillance data for all four bacteria. Although both model predictors are borderline significant in this two-predictor model, they were both significant on their own (surrounding green space; *p*‐value = 0.0165*, (log of) Euclidean distance to intact forests; *p*‐value = 0.0037**). Including both predictors brought the AIC score down four points from a single predictor model.

variable	estimate	s.e.	*z*-value	*p*‐value
**pathogen richness**				
surrounding green space	0.84	0.45	1.88	0.0598
(log of) Euclidean distance to intact forest	−0.26	0.13	−1.93	0.0540

### 3.6. *Rickettsia tillamookensis* nymphal infection status predicted by connectivity to intact habitat

Our *R. tillamookensis* binomial infection status model was the only model that was better predicted by distance to intact habitat versus distance to intact forests (i.e. only conifer and/or hardwood habitat), which was nearly significant (estimate = −0.58, *p*‐value = 0.0708; table 2). The *R. tillamookensis* NIS model was almost equally predicted by the host community cost-distance, as Euclidean distance to intact habitats (estimate = −0.50, *p*‐value = 0.0712).

### 3.7. *Borrelia miyamotoi* nymphal infection status predicted by host community cost-distance to intact forest

*Borrelia miyamotoi* infection status was solely predicted by the host community cost-distance to intact forest. As cost-distance went up, *B. miyamotoi* NIS decreased (estimate = −0.19; table 2). This was the only model that was predicted by one of our cost-distance metrics, but none of our Euclidean distance metrics (nor any other landscape or wildlife metrics).

## 4. Discussion

The distribution of tick-borne pathogens across a fragmented landscape depends on the density of ticks, the host community that ticks rely on for their bloodmeals, and potentially the transmission mode of the pathogen. Horizontally transmitted pathogens rely on specific suites of vertebrate hosts that are required for the tick to complete its life cycle and act as reservoirs of the pathogen itself, whereas pathogens with transovarial transmission do not need a reservoir host to maintain the pathogen but do require bloodmeal hosts as part of a successful tick life cycle. Because of these requirements, predicting the impacts of landscape patterns and habitat fragmentation on the spatial distribution of tick-borne pathogens is complex and species-specific. Here, we show that landscape patterns do indeed predict both the richness and prevalence of tick-borne pathogens depending on the pathogens’ transmission mode (tables 1–3). When we examine d several pathogens found in nymphal *I. pacificus*, we found that pathogen richness was greatest when habitats were not isolated (i.e. close to intact forests). We also found that dense surrounding green space had a positive relationship to pathogen richness and was more important than the habitat patch size itself (table 3). These results show that localized measurements of nearby or adjacent green space are the most important parameters for predicting the distribution of tick-associated bacterial communities, and more important than habitat fragment size.

Overall nymph density was highly dependent on habitat patch size and distance to intact forests; *I. pacificus* nymphal densities were greater in large habitat fragments that were less isolated from intact forests, indicating that the potential for humans to encounter ticks is higher in large preserves or sites that are close to contiguous natural areas (table 1). These findings stand in contrast to early patterns from the eastern United States, where higher densities of the blacklegged tick, *Ixodes scapularis*, were found in the smallest forest fragments, which are often correlated with high proportions of edge ecotone [5,6,14,77,78]. However, more recent research from the east coast has found either no pattern between forest fragmentation and proxies of disease risk metrics, or the inverse effect of patch size on disease risk [6,14,79,80]. For our study system, these landscape effects also applied to spatial patterns of infected ticks with *I. pacificus* exhibiting higher infection rates of *B. burgdorferi* s.l. in less isolated fragments. However, when we examined the relationship between habitat patch size and *I. pacificus* nymphs, we found that there was a positive relationship with nymphal tick density but a negative relationship with density of *B. burgdorferi* s.l. infected nymphs (table 1; figures 2a and 3a). Since most human cases of Lyme disease are transmitted via nymphal ticks, our results suggest that overall tick encounters, or acarological risk, will be greatest in a large habitat, but Lyme disease risk is highest in small and minimally isolated habitat fragments, such as local parks adjacent to open space preserves. High habitat isolation distance may lead to impermeability for new wildlife reservoirs and therefore reduce the presence of horizontally transmitted pathogens, such as *B. burgdorferi* s.l.*,* if transmission rates between tick life stages are low within the patch or if the pathogen encounters too many dead-end wildlife hosts.

Central to untangling the impacts of habitat fragmentation on tick-borne diseases is parsing out the relationship of landscape metrics to pathogens capable of transovarial transmission. Although most tick-borne pathogens rely on transstadial transmission between tick life stages for maintenance, the ability of a pathogen to be transovarially transmitted between adult female ticks and their eggs provides flexibility for the pathogen [81]. Some benefits of transovarial transmission include pathogen maintenance in the absence of competent reservoir hosts, reduced need for infections to be maintained long-term in bloodmeal hosts, and the allowance for variability in bloodmeal host type between environments. Our study looked at one pathogen capable of transovarial transmission, *B. miyamotoi*, one pathogen group that relies exclusively on horizontal and transstadial transmission, *B. burgdorferi* s.l., and one newly categorized bacterium with an unknown transmission mode, *R. tillamookensis* but is probably transovarially transmitted given its position in the transitional group of Rickettsia [29].

Interestingly, of the bacteria we evaluated, *B. burgdorferi* s.l. was the only pathogen group whose infection prevalence was significantly impacted by any model parameters, specifically wildlife diversity, rodent diversity and rodent richness (table 1). These results strongly suggest that host community composition is a critical component for predicting the transmission dynamics and risk of *B. burgdorferi* s.l., and potentially, non-vertically transmitted pathogens in general. While some studies have found that higher species diversity drives down disease risk [82], we found that wildlife diversity is positively associated with both the infection prevalence of *B. burgdorferi* and *B. burgdorferi* infection status among nymphs. This difference indicates the importance of accounting for species identity and host–pathogen competency. While the dilution effect predicts higher pathogen prevalence in habitats with low species diversity [83–85], the Lyme disease system in the western United States has several similarly competent reservoirs hosts for *B. burgdorferi* [ 56,16,86,87], a factor which seems to reverse the dilution effect here and calls for further emphasis on host identity at the patch level [88,89].

We found no significant landscape or wildlife predictors of *R. tillamookensis* infection status within questing nymphs, and only a single predictor of *B. miyamotoi* infection status (host community cost-distance to intact forest). For *B. miyamotoi*, this lack of wildlife predictors may indicate one of several things; limited dependence on reservoir hosts for maintenance owing to its complex vertical and horizontal transmission cycles, a reliance on a species group that is highly adaptive to the urban gradient, such as squirrels (*Sciurus* sp.), coyotes (*C. latrans*) or mice (*Peromyscus* sp.) and therefore would not be strongly impacted by habitat fragmentation [90–92], or a heightened reliance on species we did not measure in our study. However, because *B. miyamotoi* NIS was the only disease metric better predicted by host community cost-distance over Euclidean distance, we can infer that host community is impacting the pathogen’s distribution. This incongruity between the lack of wildlife predictors and the significance of host community connectivity may mean there is a missing piece of this puzzle for *B. miyamotoi* transmission. We know that *B. miyamotoi* has been found in larvae across our region and therefore, nymphal infections may not be the best indicator of disease risk for this species [27,32].

*Rickettsia tillamookensis* is a newly emerging potential pathogen and there is very little known about its sylvatic transmission cycle [29,52]. Although we did not find a significant relationship between *R. tillamookensis* infection and fragmentation metrics, there was a potential pattern that showed decreasing nymphal infections with increasing Euclidean distance to intact habitat (table 2, estimate = −0.58, *p*‐value = 0.070). These initial findings suggest that further studies may show *R. tillamookensis* transmission risk increases with proximity to intact habitat and may be indicative of *R. tillamookensis’* phylogenetic relatedness to carnivore-associated species such as *Rickettsia felis* [29,93], as carnivores generally require large habitats [94,95]. Our *R. tillamookensis* tick infection status model was the only model better predicted by isolation from all intact habitats rather than isolation to intact forests specifically, indicating that permeability to habitat suitable for large vertebrates might be a more important driver of *R. tillamookensis* distribution than permeability to areas suitable for the vector, *I. pacificus*. Further investigation of *R. tillamookensis* distribution patterns will help to characterize this potential novel pathogen.

**Figure 5.**
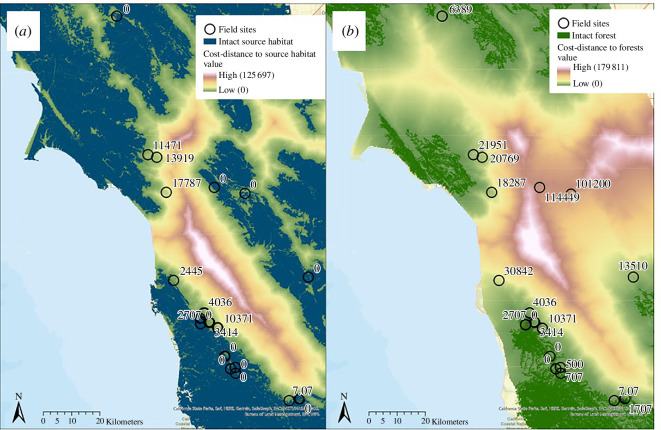
(*a*–*b*) Northern California. Map of the community-level cost-distance of sites to intact habitats and intact forests. Cost-distance values represent the permeability of the landscape to wildlife movement and dispersal. Large cost-distance values represent isolated habitat fragments. Sites that are located within intact habitat have a cost-distance value of 0.

In most cases, we found no benefit to the use of cost-distance over the simpler Euclidean distance measurements of isolation. Despite extensive wildlife movement research, the expert input of 30 mammologists, and the use of highly specific land cover data, cost-distance was often similarly or slightly less predictive of our response variables compared to Euclidean distance measurements of isolation, with the exception of *B. miyamotoi* infection status. One limitation in this analysis lies with the difficulty in accounting for dispersal-limited species. We tried to address the small-home range sizes of rodents with the more localized surrounding green space metric but even so, there is large variability in dispersal capabilities and preferences between our 15 focal species which go beyond land cover permeability and should be considered in future studies. However, we would caution others to review the details of our study system before discounting the application of cost-distance tools for future pathogen surveillance or entomological research. Our study area is more than six times the size of Antwerp, Belgium, where previous cost-distance analysis showed correlations with infected ticks in the Lyme disease system [11]. The large distance between some of our sites and the nearest intact habitats may have diluted the nuances of fine-scale wildlife movement patterns (figure 5*a,b*). Cost-distance may be a more important tool when used at a smaller scale or within denser, urban landscapes. Additionally, it is important to note that alternative connectivity tools such as circuit modelling and resistant kernels are available. These may provide benefits over cost-distance analysis as they can model a range of likely pathways at once, do not require the input of destination points and can sometimes account for dispersal limited species [39,40,42]. Other vector ecology studies have found important impacts of habitat stratification [96–98], landscape disturbance by invasive plant species [99,100] and the type of transition zones between habitat and matrix [101,102]. Owing to the scale of this study, we did not explicitly measure these variables. Further exploration of these factors may shed light on additional nuances of habitat composition.

Our findings highlight the importance of studying landscape ecology to understand pathogen distribution, especially for vector-borne disease systems which involve many mitigating factors, including reservoir host availability. Landscape variables proved predictive of DON, DIN, NIS and pathogen richness, while wildlife parameters helped to explain only a subset of these metrics. These data support the application of island biogeography theory to co-occurring vector-borne disease systems in a ‘mainland island’ environment, where we find a higher diversity of pathogens in patches surrounded by more green space and those that are less isolated compared to small and physically distant habitat ‘islands’.

The results of this study suggest that city layouts with more isolated green spaces would prove beneficial to reducing human Lyme disease risk. However, human tick-borne disease risk is only one facet of habitat connectivity to consider when planning urban development. Human and environmental health, as well as biodiversity priorities, must also be accounted for. In an era of increasing human–wildlife interfaces, it is imperative to track and predict how landscape modifications impact vector-borne disease risk under distinct ecological contexts. We have shown here that targeted surveillance of vector-borne diseases in minimally isolated habitats can provide efficient and focussed determinations of disease risk in urban areas.

## Data Availability

The data that support the findings of this study are openly available on Dryad [103]. Sequences identified to species level have been submitted to GenBank (Accession numbers PQ315998—PQ316045, PP707645—PP707660, PQ375123—PQ375160, and PP707690—PP707719). Supplementary material is available online [104].
